# Participation of Polymer Materials in the Structure of Piezoelectric Composites

**DOI:** 10.3390/polym16243603

**Published:** 2024-12-23

**Authors:** Cosmin Ionuț Pîrvu, Alexandru Sover, Mărioara Abrudeanu

**Affiliations:** 1 Doctoral School of Materials Science and Engineering, National University of Science and Technology POLITEHNICA Bucharest, Splaiul Independenței nr. 313, Sector 6, 060042 Bucureşti, Romania; 2 Institute for Nuclear Research, Câmpului Street nr. 1, 115400 Mioveni, Romania; 3 Faculty of Engineering, ANSBACH University of Applied Sciences, Residenzstraße 8, 91522 Ansbach, Germany; a.sover@hs-ansbach.de; 4 Technical Sciences Academy of Romania, Calea Victoriei nr. 118, Sector 1, 010093 Bucuresti, Romania

**Keywords:** piezoelectric, lead-free ceramics, polymers

## Abstract

This review explores the integration of polymer materials into piezoelectric composite structures, focusing on their application in sensor technologies, and wearable electronics. Piezoelectric composites combining ceramic phases like BaTiO_3_, KNN, or PZT with polymers such as PVDF exhibit significant potential due to their enhanced flexibility, processability, and electrical performance. The synergy between the high piezoelectric sensitivity of ceramics and the mechanical flexibility of polymers enables the development of advanced materials for biomedical devices, energy conversion, and smart infrastructure applications. This review discusses the evolution of lead-free ceramics, the challenges in improving polymer–ceramic interfaces, and innovations like 3D printing and surface functionalization, which enhance charge transfer and material durability. It also covers the effects of radiation on these materials, particularly in nuclear applications, and strategies to enhance radiation resistance. The review concludes that polymer materials play a critical role in advancing piezoelectric composite technologies by addressing environmental and functional challenges, paving the way for future innovations.

## 1. Introduction

**Figure 1:**
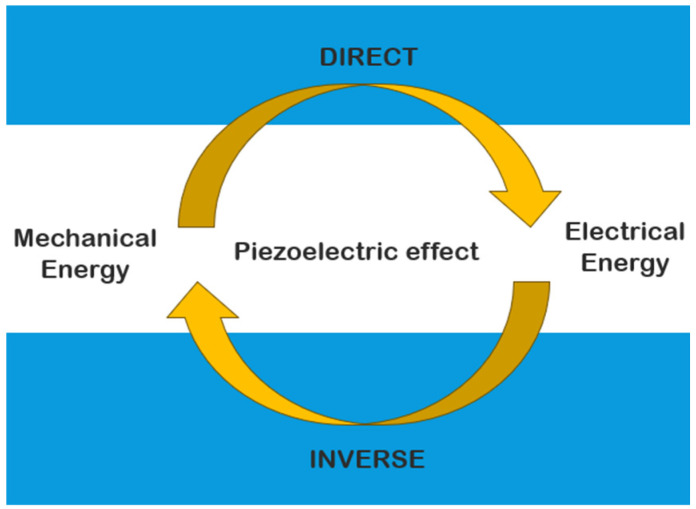
Piezoelectric effect, after [3].

Piezoelectric materials are widely used in various applications, from sensors and actuators to energy harvesting systems. These materials possess the unique ability to convert mechanical energy into electrical energy and vice versa (Figure 1), making them of interest in fields like energy generation, sensors, actuators, and other applications. Piezoelectric ceramics such as barium titanate (BaTiO_3_) and lead zirconate titanate (PZT) offer high piezoelectric coefficients, but they are brittle and difficult to integrate into flexible systems. Recent research has focused on developing hybrid piezoelectric composites with remarkable piezoelectric properties and structural flexibility, combining the high piezoelectric properties of ceramic materials with the flexibility and processability of polymers [1,2,3,4].

**Figure 2:**
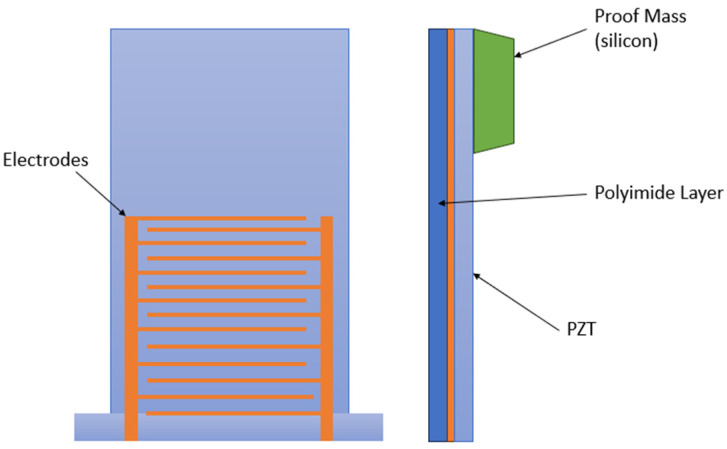
Flexible PZT device for energy harvesting, after [5].

Research on hybrid piezoelectric materials has significantly advanced in the past decade due to the development of niche applications such as biomedical devices and environmental energy harvesting systems (Figure 2) [5,6,7].

Polymer–ceramic piezoelectric composites are widely used in flexible devices, wearable electronics, and smart infrastructures. Polymers play an essential role in these composites, providing flexibility and better processability, while the ceramic phase contributes to high piezoelectric response. Additionally, molecular-level interaction between polymer and ceramic is crucial for the overall performance of the composites [8,9].

As the demand for flexible and wearable devices increases, developing piezoelectric composites with improved mechanical and electrical properties becomes increasingly important. Hybrid materials that combine the advantages of each component (polymer and ceramic) are considered key for the development of new technologies in sensors, energy harvesting, and biomedical devices [10,11,12].

In modern devices, their role extends beyond traditional uses in ultrasonic sensors and transducers to encompass emerging technologies. For instance, in energy harvesting systems, piezoelectric composites have been successfully implemented in self-powered sensors for structural health monitoring. One notable example is the piezoelectric energy harvester embedded in aircraft wings to monitor structural integrity during flight, reducing maintenance costs and improving safety [13,14,15].

In wearable electronics, piezoelectric materials have revolutionized real-time health monitoring. Flexible sensors based on polyvinylidene fluoride (PVDF) composites have been integrated into smart clothing to measure physiological signals such as heartbeat, respiration, and motion. A prominent example is the Smart Sock [16], which uses piezoelectric sensors to track foot pressure patterns, providing valuable insights for athletes and individuals undergoing rehab [17].

Moreover, piezoelectric actuators play a critical role in precision engineering. In robotics, piezoelectric-driven micro-actuators are employed in surgical robots like the Da Vinci Surgical System [18], enabling highly precise and minimally invasive operations. In micro-electromechanical systems (MEMSs), piezoelectric materials are used in inkjet printers to control the ejection of ink droplets with exceptional precision and speed, and in autofocus mechanisms in smartphone cameras for enhanced image clarity.

### Proposed Methodology

The aim of this review is to analyze the participation of polymeric materials in the structure of piezoelectric composites, their integration into devices, energy conversion technologies, and smart infrastructures. This may include identifying the advantages and limitations of ceramic–polymer hybrid materials and employing techniques such as 3D printing and surface functionalization.

- The scope focuses on the applications of piezoelectric materials in sensors and portable electronics, energy regeneration, smart infrastructures, nuclear energy, and environmental applications.
- The role of the review is to synthesize and organize existing data, highlighting the evolution of lead-free piezoelectric materials, solutions for improving polymer–ceramic interfaces, theoretical models for charge transfer, and the effects of radiation on composite performance.
- The author’s intention is to identify sustainable and efficient solutions for the development of piezoelectric technologies, tailored to modern requirements and environmental regulations, while promoting eco-friendly and durable innovations.

## 2. Ceramic Phases

The most common material is lead zirconate titanate Pb(Zr,Ti)O_3_ (PZT), being one of the most used piezoelectric materials due to its high piezoelectric coefficients (ranging from 300 to 700 pC/N) and excellent high-temperature stability. This makes it ideal for applications requiring strong electrical signals under mechanical stress. However, the presence of lead makes this material toxic, and its use has been regulated by European directives since 2002 [19,20,21], prompting research into lead-free alternatives [22] like potassium niobate (KNbO_3_), sodium potassium niobate (KNN), and barium titanate (BaTiO_3_) [23,24,25,26]. So far, no material has fully matched the functional properties of PZT for the wide range of piezoelectric applications in which it is implemented.

Barium titanate, BT (BaTiO_3_), was the first discovered polycrystalline ceramic material to exhibit ferroelectricity, being considered a serious candidate for piezoelectric transducer applications. PZT, discovered later, shows better piezoelectric properties than BT and has a higher Curie temperature, which reduced the interest in using BT for piezoelectric applications. Subsequent research on BT doped with various elements has led to a reconsideration of the potential of BT-based materials for piezoelectric applications.

In the work by Song, Xuan, and collaborators [27], a summary of the evolution of knowledge about BT is presented, from its independent discovery in the United States, the United Kingdom, and Russia, to its simpler crystalline structure, with a displacement-type phase transition at the Curie temperature, and up to the current technological potential of BT-based materials for piezoelectric applications.

**Figure 3:**
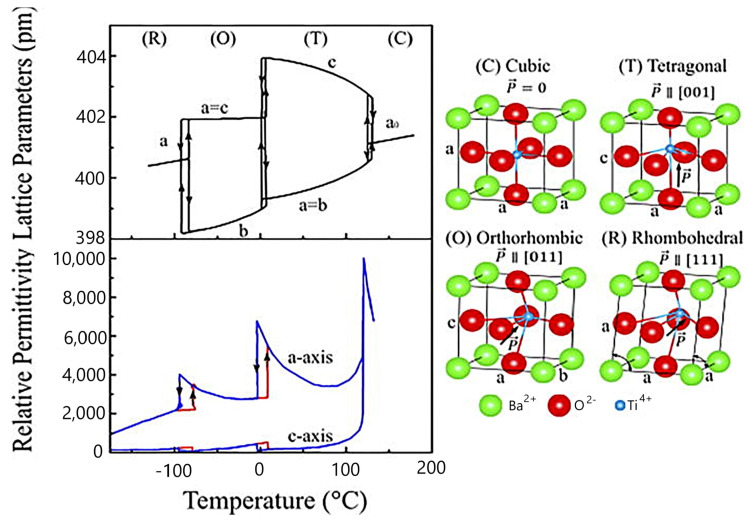
Structure properties of BT, after [28].

Barium titanate belongs to the perovskite family with the general formula ABO_3_. The Ba^2+^ ion is located in position A at the corners of the cubic unit cell, while the Ti^4+^ ion occupies position B at the center of the cell. The O^2−^ anions are located at the centers of the faces of the unit cell, forming octahedra (Figure 3). The appearance of spontaneous polarization in barium titanate is explained crystallographically as being related to a displacement of the Ti^4+^ and O^2−^ ions relative to the Ba^2+^ ion at the origin. The resulting electric dipole moment is known as spontaneous polarization. The appearance of spontaneous polarization is accompanied by changes in the dimensions of the unit cell. The deformation relative to the cubic prototype due to these dimensional changes is called spontaneous strain [28].

The cubic phase of barium titanate belongs to the class of centrosymmetric crystals; it is paraelectric and non-piezoelectric. The order parameter for the transition from the cubic prototype phase to the ferroelectric phases with lower symmetry is spontaneous polarization. The prototype phase of perovskite crystals allows for seven suitable ferroelectric phases: one tetragonal phase, one rhombohedral phase, one orthorhombic phase, three monoclinic phases, and one triclinic phase.

The transition between the cubic and tetragonal structures is a paraelectric–ferroelectric transition that leads to the appearance of spontaneous polarization. In barium titanate, this transition is of first order. Above the Curie temperature, the dielectric susceptibility follows the Curie–Weiss law. Lower temperature transitions involve a reorientation of the polarization direction and are often referred to as inter-ferroelectric transitions. The tetragonal ↔ orthorhombic and orthorhombic ↔ rhombohedral transitions are also first-order transitions.

BT (barium titanate) is a basic piezoelectric material commonly used due to its exceptional performance at room temperature and ease of fabrication. Recently, advances have been made in the manufacturing of BaTiO_3_ composites with polymers that demonstrate a significant increase in piezoelectric sensitivity, having potential applications in electronics and sensor technology. This material features a perovskite structure and is characterized by its stability in moderate electric fields and varying temperatures [29,30].

BaTiO_3_-based composites are of particular interest for environmentally sensitive applications, such as pressure sensors and energy harvesting devices [31,32]. Additionally, BaTiO_3_ is often used in combination with flexible polymers such as PVDF to create piezoelectric composites capable of functioning in flexible and dynamic environments [33,34,35,36].

Methods for synthesizing barium titanate are widely discussed in the literature. The choice of synthesis technique is influenced not only by costs but also by the specific final application. The synthesis process and the quality of the starting materials affect the final properties of the powder. As electronic devices continue to be miniaturized, the demand for powders with smaller particle sizes and precise morphology becomes critical. The purity and crystalline structure of BaTiO_3_ are essential factors influencing its dielectric properties, with each synthesis method having specific benefits. The main synthesis methods used in practice are presented as follows [37,38]:

- Conventional solid-state reaction;
- Chemical methods for the synthesis of barium titanate;
- Sol–Gel method;
- Hydrothermal method;
- Coprecipitation method;
- Polymer precursor method;
- Mechanochemical synthesis.

**Figure 4:**
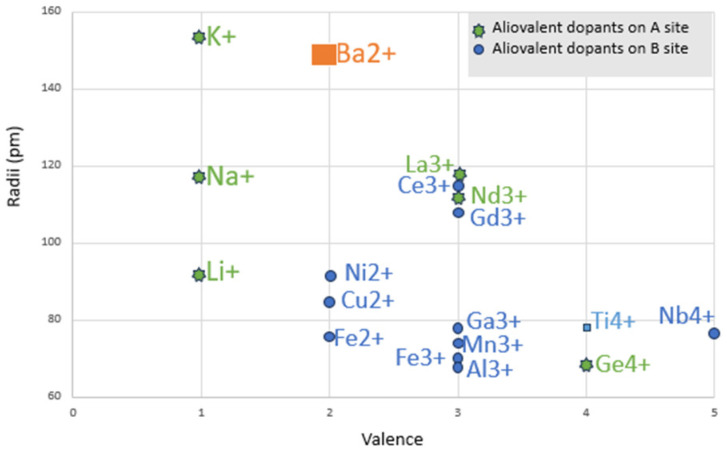
BaTiO_3_ main dopants, after [39].

To achieve superior properties such as electrical, mechanical, or structural characteristics of the material, modifications of the Curie temperature, thermal stability, or piezoelectric constants, the practice of doping barium titanate with various dopants is common. This can introduce defects into the lattice or modify the behavior of ions. The main dopants used for barium titanate are shown in Figure 4.

Doping barium titanate is a key strategy for improving its piezoelectric performance [40]. Depending on the nature of the dopant (donor, acceptor, or isovalent), changes can be achieved in the dielectric constant, the stability of ferroelectric domains, the Curie temperature, and the piezoelectric constants. Dopants provide additional flexibility in tailoring the material to meet the requirements of specific applications [41].

Potassium niobate (KNbO_3_) has attracted attention for its piezoelectric coefficient compared to PZT, with the added benefit of being non-toxic, making it an attractive alternative in the context of increasingly strict environmental regulations. Recent research suggests that doping additives, such as Li⁺ or Na⁺, can significantly improve the piezoelectric performance of KNbO_3_ [42].

Sodium potassium niobate (KNN) is another promising material that has been intensively studied. Recent studies have shown that its chemical structure allows for greater customization of piezoelectric properties through composition modifications, such as ionic substitution or the addition of other ceramic phases [43]. KNN is also recognized for its thermal stability and its ability to function efficiently in various environmental conditions. The piezoelectric properties of these phases can be considerably improved by refining the structure and using dopants.

## 3. Structures of Polymer–Ceramic Piezoelectric Composites

**Figure 5:**
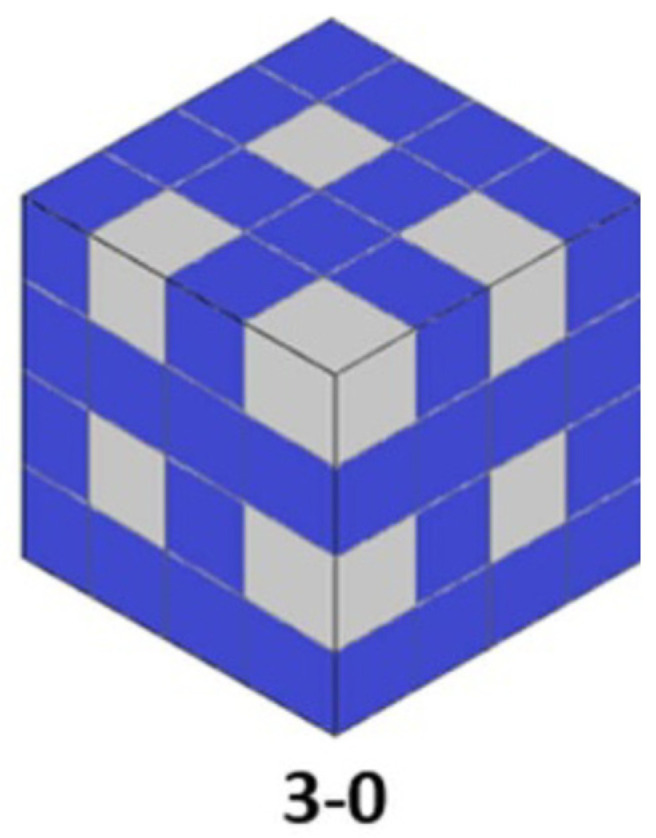
A 3-0 composite structure, after [5].

Polymer–ceramic composites with 0–3 geometry are among the most common structures used in piezoelectric composites (Figure 5). In these materials, piezoelectric ceramic particles are uniformly distributed in a polymer matrix, forming a dispersed network. Although their simple structure and manufacturing flexibility make them attractive, uneven dispersion of ceramic particles can limit piezoelectric performance. Recent improvements have focused on optimizing particle morphology and using coupling agents and chemical modifiers to enhance compatibility between the ceramic and polymer phases. It has been shown that the use of coupling agents, such as conductive nanofillers (like graphene or carbon nanotubes), can significantly improve piezoelectric performance by increasing relative permittivity and reducing dielectric losses [44,45,46].

Studies on the development of lead-free hybrid materials, using sodium potassium niobate (KNN) or barium titanate (BaTiO_3_), have shown that comparable performance to lead-based materials can be achieved, along with ecological benefits. These materials, combined with UV-curable polymers, have enabled the development of thin and flexible composites with applications in sensors and structural monitoring [47].

By controlling the morphology and size of ceramic particles, considerable improvements in the piezoelectric and mechanical properties of 3-0 composites can be achieved. Optimizing the distribution and orientation of the particles in the polymer matrix results in a significant increase in the piezoelectric coupling factor and the dielectric constants of the composite [48]. The quality of the ceramic insert–polymer matrix interface, improved by the addition of coupling agents (e.g., silanes), allows for increased charge transfer and overall material performance. The development of piezoelectric composites with ceramic nanoparticles, with a high specific surface area, has allowed for increased interaction between the ceramic and polymer.

### 3.1. Polymer/Ceramic Filler Adhesion Mechanism

The adhesion mechanism between the polymer matrix and ceramic filler plays a pivotal role in determining the piezoelectric performance of the composite. Strong interfacial bonding ensures efficient stress transfer from the polymer to the ceramic phase, thereby enhancing the overall piezoelectric response [49].

### 3.2. Interaction Mechanism at the Interface

The interaction at the polymer/ceramic interface can be mechanical, chemical, or electrostatic. Mechanical interlocking occurs when the surface morphology of the ceramic filler provides physical anchoring points for the polymer chains. Electrostatic interactions, such as dipole–dipole forces, are significant when polar polymers or functional groups interact with charged surfaces on the ceramic fillers [50].

### 3.3. Common Functionalization Strategies

Chemical functionalization is often employed to enhance interfacial bonding [51]. Commonly used chemical groups include the following:

- Silane Coupling Agents: Functional groups such as aminosilanes and epoxysilanes are grafted onto ceramic surfaces to create covalent bonds with polymer matrices, promoting strong adhesion [52].
- Carboxyl and Hydroxyl Groups: These groups, introduced via surface treatments like plasma or acid etching, improve hydrophilicity and chemical compatibility with polar polymer matrices [53].
- Ionic Liquids and Surfactants: These can modify the surface charge of ceramics, improving dispersion and interaction with polymer matrices [54].

### 3.4. Role at the Interface

These chemical modifications reduce the interfacial energy, facilitating better stress transfer across the polymer/ceramic boundary. Improved adhesion minimizes the formation of interfacial voids and defects, which can detract from the piezoelectric performance by causing local stress concentration and reducing charge transfer efficiency [55].

The use of processing techniques such as 3D printing and injection molding allows for better control of the composites and reduces internal defects that limit piezoelectric efficiency [56].

These advances lead to the development of higher-performance materials used for a wide range of applications, such as actuators and energy harvesting devices.

The component materials, through their properties and characteristic structure, determine the composite’s properties and areas of application. The 3-0 composites made of dispersed PZT (lead zirconate titanate) particles in an epoxy or silicone polymer matrix exhibit moderate electrical properties and good flexibility, making them suitable for pressure and vibration sensors, smart materials, and energy harvesters.

The composite made from BaTiO_3_ particles in a PMMA (polymethylmethacrylate) or PVDF (polyvinylidene fluoride) matrix, with good dielectric and piezoelectric properties and easy processing, is used in capacitors, microelectronics, and energy harvesting devices.

Material made from ZnO particles dispersed in a polymer matrix (polyurethane) can be used to create flexible sensors that are easy to integrate into complex structures, such as tactile sensors, flexible displays, and actuators for portable devices.

Lithium niobate (LiNbO_3_) in a PDMS (polydimethylsiloxane) polymer matrix is a high-performance material used in applications requiring precise piezoelectric properties and good optical transparency: optical sensors, actuators, and in optoelectronic technology.

**Figure 6:**
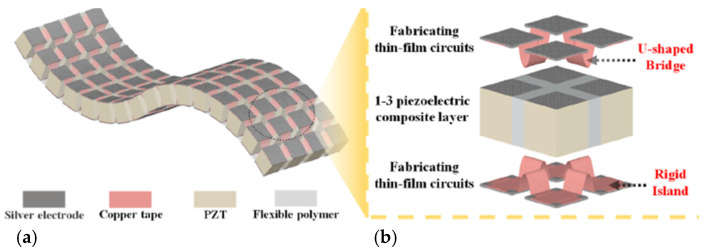
Composite type 1-3: (**a**) structure of 1-3 composite; (**b**) flexible 1-3 composite [61].

Polymer–ceramic composites with 1-3 geometry are made of continuous piezoelectric fiber in a non-piezoelectric matrix. This structure offers good directionality of piezoelectric properties. The piezoelectric components, in the form of fibers or filaments, are aligned and distributed in a continuous polymer matrix (Figure 6a).

The term “1-3” refers to the fact that the components are continuous in one direction (1 dimension of connectivity), while the matrix is continuous in the other three directions (3 dimensions of connectivity). This structure allows for more efficient generation of electrical charge along a directional axis [57,58]. These materials are used in applications requiring directional piezoelectric response, such as vibration sensors or precision actuators [59]. Studies have shown that fiber alignment in the desired direction significantly influences piezoelectric efficiency and charge generation capacity.

Additionally, the use of flexible polymers in 1-3 composites significantly improves the materials’ flexibility and durability, allowing their use in applications where flexibility is essential, such as robotics or in the manufacturing of medical instruments (Figure 6b). The 1-3 composites offer both advanced technical performance and versatility, making them a popular choice in modern industry [60,61,62,63].

**Figure 7:**
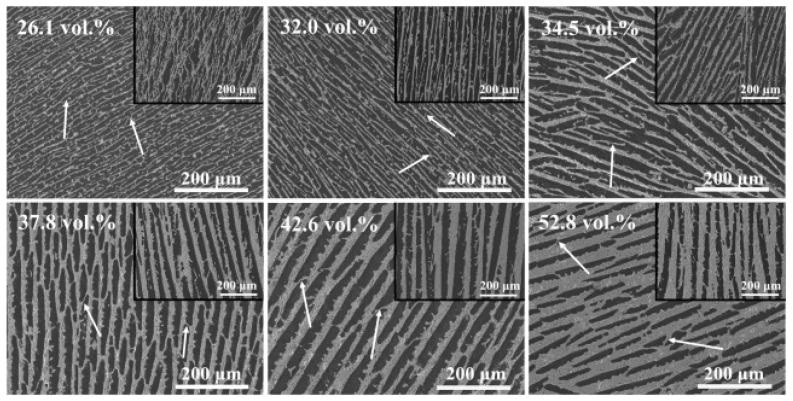
Cross-sectional morphologies of BaTiO_3_/PMMA 1-3 bio-piezoelectric composites with different BaTiO_3_ contents (after Yufei Tang and collaborators) [64].

Yufei Tang and collaborators [64], using directional freeze casting and the self-solidification of bone cement, obtained BaTiO_3_/PMMA 1-3 bio-piezoelectric composites with a lamellar structure (Figure 7). The lamellar BaTiO_3_ structure gives the composite enhanced piezoelectric properties. The dendritic ceramic bridges on the walls of the BaTiO_3_ pores can improve the compression strength and elastic modulus of the BaTiO_3_/PMMA bio-piezoelectric composites with a lamellar structure. It was found that this structure favors growth in the direction of the BaTiO_3_ layers.

The images inserted in Figure 7 show the longitudinal morphologies of the composite. The dark areas are PMMA, while the bright areas are BaTiO_3_. The white arrow indicates the dendritic morphology (protrusions and bridges). The scale bar is 200 μm.

**Figure 8:**
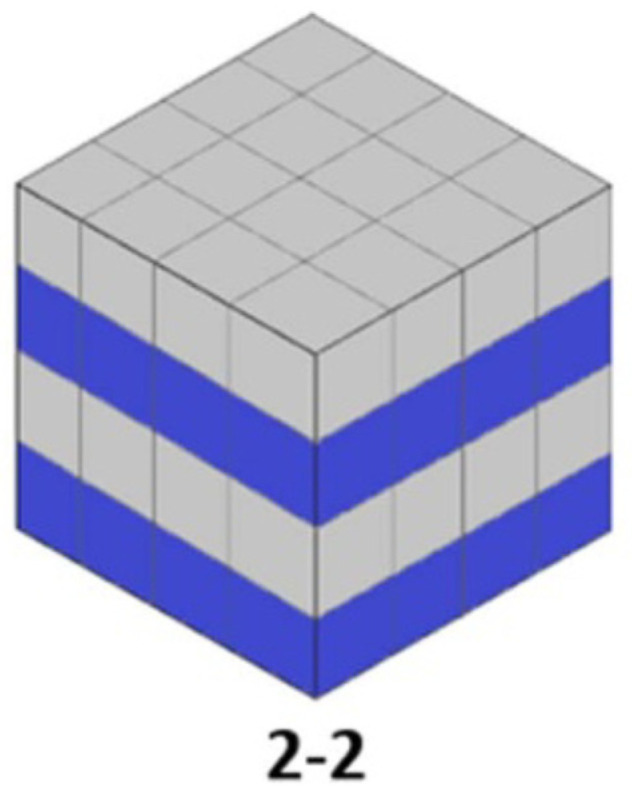
Structure of the 2-2 composite, after [5].

In the case of 2-2 layered composites, the polymers and ceramics are arranged alternately in layers (Figure 8). This configuration allows for good control over the piezoelectric properties of the material. The polymer plays a crucial role in mitigating mechanical stresses, ensuring properties of flexibility and shock resilience, adhesion, and interface stability [65].

Layered composites exhibit directional piezoelectric properties due to the one-dimensional connectivity of the piezoelectric fibers; these composites have strongly directed piezoelectric properties. The use of coupling agents leads to increased efficiency and durability. They are ideal for applications involving the detection or generation of energy in a specific direction and show good performance at low frequencies, suitable for detection applications, actuators, and energy conversion technologies [66,67,68].

## 4. Polymer Phases

Polymeric phases, depending on their nature, provide flexibility, the ability to absorb and distribute stress, high adhesion, resistance to harsh environments, transparency, or impact resistance.

Polymeric materials can be used as piezoelectric inserts or as a matrix. Piezoelectric polymers exhibit long, flexible molecular chains that allow deformation under mechanical stress. Polarization occurs when these chains are aligned through stretching or the application of an electric field. The piezoelectric properties of polymers are lower compared to ceramics, but they offer the advantages of superior flexibility and processability [69].

Among polymers with piezoelectric properties, polyvinylidene fluoride (PVDF) and its copolymers are the most widely used, due to their relatively high piezoelectric coefficients and chemical stability. Recent studies show that PVDF can achieve piezoelectric coefficients of up to 33 pC/N under certain processing conditions, making it a key material for piezoelectric applications [70]. Additionally, its crystalline structure can be controlled through processing techniques, such as mechanical orientation and thermal treatment, which allows for the improvement of piezoelectric performance [71].

The matrix plays the role of embedding the inserts, keeping them in a fixed position, distributing the loads between the inserts, and protecting them from external factors (impact, humidity, chemicals). It ensures structural cohesion and allows the creation of composite material components in complex shapes.

Polyamide (PA) and polytetrafluoroethylene (PTFE) are often used in combination with ceramic materials to improve the mechanical properties and processability of composites. Polyamide provides a high degree of tensile strength, durability, impact resistance, and flexibility—important properties in applications that require adaptation to stress variations. Recent research has shown that the addition of polyamide to ceramic–polymer composites can significantly improve mechanical and piezoelectric performance [72]. Polytetrafluoroethylene (PTFE), known for its excellent electrical insulation properties and chemical resistance, is often used to improve the thermal stability and corrosion resistance of composites. The addition of PTFE to composites also helps reduce friction losses, improving the overall performance of the system in industrial applications.

### 4.1. The Role of Polymers in the Performance of Piezoelectric Composites

Ceramic–polymer composites are designed to combine the best characteristics of both phases:

- Enhanced piezoelectric properties: This is through the synergy between the ceramic phase, which ensures electrical sensitivity, and the polymer phase, which provides flexibility and processability [73].
- Flexibility: Polymers give the composites increased malleability, allowing their use in applications where rigid ceramics could not be utilized [7].
- Mechanical durability: Polymers add impact resistance, countering the brittleness of ceramics [74].

### 4.2. Functionalization of Polymers

Polymers play a dual role in piezoelectric composites, providing both mechanical flexibility and dielectric properties essential for modulating the electrical performance of the material [75]. A critical aspect is the functionalization of polymers to enhance the interface with the ceramic phases. This involves chemical treatments to increase adhesion at the molecular level and facilitate charge transfer [76,77,78].

Chemical modifications: Chemical treatments may include the addition of reactive functional groups, such as amino or carboxyl groups, which can create stronger chemical bonds between the polymer and ceramic. These modifications can lead to a significant improvement in interfacial adhesion, which is essential for the efficiency of charge transfer and the mechanical stability of the composites.

Use of coupling agents: Coupling agents, such as silanes, can be used to improve the compatibility of polymers with ceramics. These substances form an intermediate layer that facilitates interactions between the two phases, thereby increasing charge transfer efficiency and reducing the risk of delamination.

Co-polymerization: Co-polymerization with other substances can create hybrid materials with improved properties. For example, the co-polymerization of PVDF with other polymers, such as polyethylene glycol (PEG), can enhance the processability and piezoelectric properties of the final composite.

Nanomaterials: Integrating nanomaterials into the polymer structure can lead to significant improvements in piezoelectric properties. Silver or graphene nanoparticles can increase electrical conductivity and mechanical strength, facilitating molecular-level electrical interactions and enhancing the efficiency of piezoelectric composites.

The functionalization of polymers is essential for maximizing the potential of piezoelectric ceramic–polymer composites. Through chemical treatments and the use of coupling agents, the interface between polymers and ceramics can be significantly improved, resulting in materials with superior electrical performance and increased stability.

### 4.3. Dielectric Properties

The dielectric properties of the polymer directly influence the efficiency of electric charge generation. Polymers with high dielectric constants, such as polyvinylidene fluoride (PVDF), are preferred to maximize the piezoelectric performance of the composites. PVDF is distinguished by a relatively high dielectric constant, ranging from 6 to 13, depending on its crystalline form. This characteristic allows for greater accumulation of electric charge in the presence of an applied electric field, significantly contributing to the overall efficiency of the piezoelectric material [79,80].

Polyvinylidene fluoride (PVDF) is a versatile polymer known for its piezoelectric and ferroelectric properties, which stem from its ability to adopt different crystalline phases. Among these, the β, γ, and δ phases exhibit spontaneous polarization, making them particularly relevant in advanced applications [81,82,83,84].

- The β-phase is the most significant for piezoelectric and ferroelectric applications due to its high net dipole moment. In this phase, PVDF chains adopt an all-trans (TTTT) conformation, leading to the alignment of fluorine and hydrogen atoms in opposite directions, resulting in strong polarization. Methods such as mechanical stretching, poling under electric fields, or incorporating nanoparticles like BaTiO_3_ are used to enhance the β-phase content. This phase is integral in applications like energy harvesting and sensors due to its superior piezoelectric performance.
- The γ-phase features a mixed conformation (TGTG’) and exhibits spontaneous polarization, although it is less pronounced compared to the β-phase. It provides a balance between piezoelectric properties and structural stability. The γ-phase is often encountered in PVDF blends and composites, contributing to flexibility and moderate piezoelectric responses, making it suitable for applications like flexible electronics and lightweight sensors.
- The δ-phase arises from the poling of the non-polar α-phase under high electric fields. While structurally similar to the α-phase (TGTG conformation), the dipoles in the δ-phase align to produce net polarization. The δ-phase has lower piezoelectric properties than the β-phase but remains relevant for dielectric applications and as an intermediate phase in specific processing techniques.

Additionally, the incorporation of nanostructured fillers, such as carbon nanotubes, can enhance the dielectric properties of the polymer matrix and increase charge transfer efficiency. The use of carbon nanotubes (CNT) in ceramic–polymer composites has been widely investigated, as they offer not only excellent electrical conductivity but also superior thermal and mechanical stability [85].

### 4.4. Advantages and Disadvantages

Table 1 illustrates how polymer/ceramic composites combine the advantageous properties of their constituents, such as enhanced piezoelectric and dielectric performance compared to the polymer matrix alone, and improved flexibility and processability compared to ceramics. However, trade-offs include a reduction in piezoelectric performance compared to pure ceramics and moderate thermal stability constrained by the polymer component.

Advantages

- Mechanical flexibility: Polymers allow for the integration of composites into flexible applications, such as portable electronics or biomedical implants [7,86,87,88,89,90,91,92,93].
- Processability: Polymers enable the fabrication of composites in complex shapes through techniques such as injection molding or casting [94,95].
- Lightweight: Compared to fully ceramic materials, composites have a lower density [95].

Disadvantages

- Decrease in piezoelectric coefficients: The addition of a polymer phase tends to reduce the overall piezoelectric performance of the composite compared to fully ceramic materials [85,96].
- Trade-off between flexibility and rigidity: Increasing the proportion of the polymer phase can lead to a reduction in mechanical stability and piezoelectric coefficients [86].

## 5. Manufacturing and Processing Methods

Traditional techniques for manufacturing piezoelectric composites through sintering and hot pressing [97,98] are not suitable for composites with polymers that cannot withstand high temperatures. Among alternative processes, casting and extrusion can be used to obtain polymer–ceramic composites [99,100,101].

Three-dimensional printing has become a revolutionary method in the fabrication of piezoelectric composites, providing flexibility in the design and manufacturing of complex materials, as well as the possibility to integrate different phases in a controlled manner [102]. Direct Ink Writing (DIW) technology enables the precise deposition of piezoelectric materials onto flexible substrates (fabrics) and their integration into wearable electronics [103,104,105]. Due to its high precision and ability to deposit piezoelectric inks with customized properties, the DIW technique is especially used for the fabrication of pressure sensors and actuators [106].

The limitations and constraints associated with selecting 3D printing techniques [107,108] for manufacturing piezoelectric components can be analyzed based on the following factors:

- Material compatibility: Not all 3D printing techniques can handle piezoelectric ceramic materials or their composites (e.g., PZT ceramics). Techniques such as stereolithography (SLA) are better suited for piezoelectric materials due to their precision and ability to work with ceramic suspensions.
- Resolution and geometry of parts: High-precision techniques, such as photopolymerization-based printing, are more efficient for achieving complex details.
- Mechanical and electrical properties: The density, microstructure, and piezoelectric performance of the material are determined by the printing method, which can affect the piezoelectric performance. Post-printing processing (e.g., sintering, poling) is essential to restore piezoelectric properties.
- Size and production scale: Three-dimensional printing is limited for mass production due to its relatively slow speed and high costs. It is recommended primarily for prototyping or manufacturing customized components.
- Integration of piezoelectric composites: Creating piezoelectric composites that include polymeric materials can be challenging depending on the technique used. Extrusion-based or multi-material printing provides flexibility for integrating polymers and ceramics.

**Figure 9:**
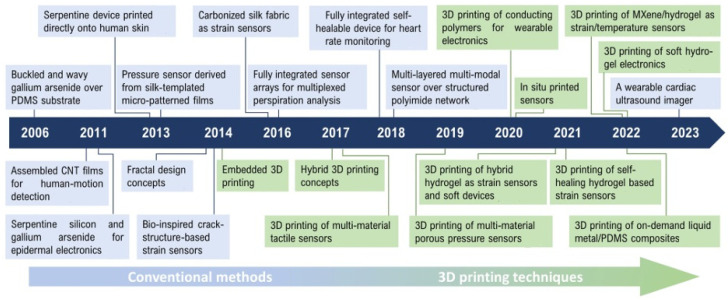
Summary of recent studies on the manufacturing of wearable sensors using conventional techniques and 3D printing (according to Amr Osman and Jian Lu) [109].

Amr Osman and Jian Lu provide a synthesis of the evolution of manufacturing techniques for wearable sensors, from conventional techniques to 3D printing from 2006 to 2023 (Figure 9) [109].

**Figure 10:**
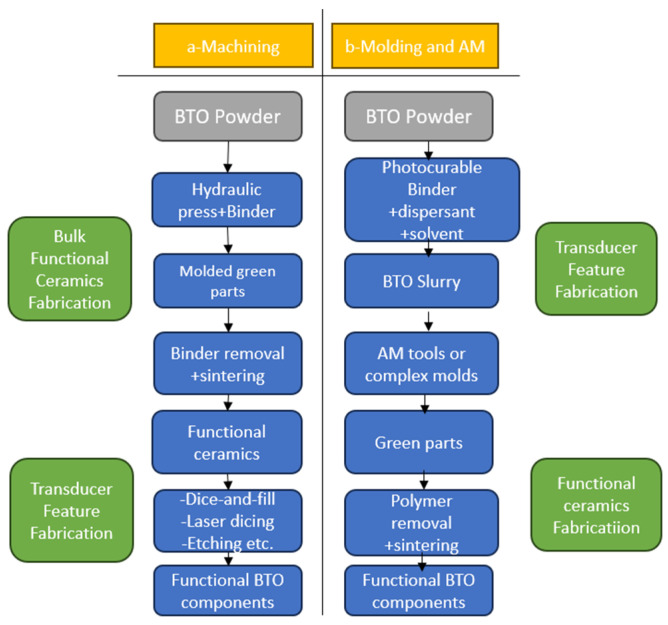
Manufacturing of piezoelectric composites: (**a**) classical methods; (**b**) additive manufacturing (after Xuan Song and collaborators) [27].

Song, Xuan, and collaborators make a comparison regarding the manufacturing of piezoelectric components through classical methods (see Figure 10a) and additive manufacturing (see Figure 10b) [27]. In these approaches, the piezocomposite suspension is first prepared by mixing piezo-ceramic powders with polymers and solutions in specific mixing ratios.

In the paper [110], Cheng Chen and collaborators present that recent advances in additive manufacturing of piezoelectric materials have been significant, highlighting the advantages of 3D printing technology and its technical impact on the production of these materials. Possible future development directions in additive manufacturing research have been identified, as well as the current limitations of this technology in the piezoelectric materials industry.

**Figure 11:**
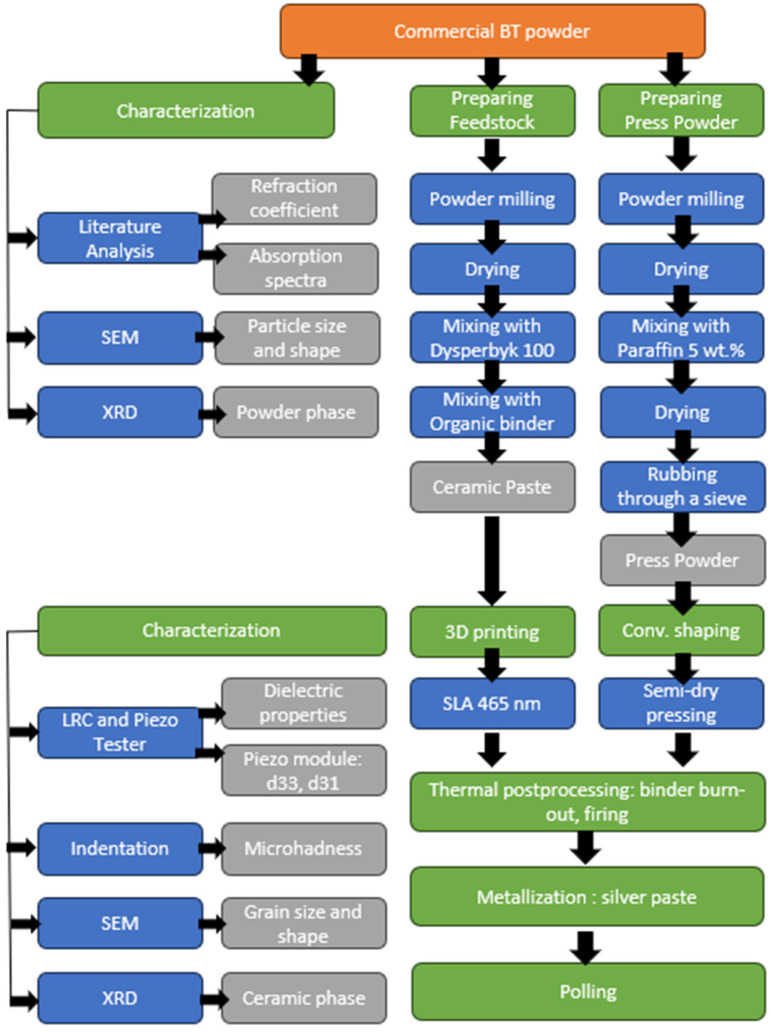
Comparative diagram of the behavior of BT powder in conventional and SLA-based additive manufacturing processes (after Andrey Smirnov) [111].

Anrey Smirnov and collaborators conducted a comparative experimental study on the behavior of BT powder in conventional and additive manufacturing processes based on SLA [111]. They observed that, in the case of additive manufacturing, the steps taken to achieve the final part were fewer than in conventional manufacturing. This can be seen in Figure 11.

Stereolithography (SLA) allows for the printing of piezoelectric structures with very high resolution and improved mechanical properties. SLA uses a laser to solidify photopolymer resins, enabling the creation of complex piezoelectric composites with precise layered structures [110,112,113]. This method is ideal for the development of biomedical devices and small-sized sensors [109]. Recently, SLA has been used for the fabrication of piezoelectric transducers and energy harvesting devices [114,115].

## 6. Examples of Ceramic–Polymer Composites and Their Characterization

### 6.1. PZT-PVDF Composites

A classic example of a composite is the combination of lead zirconate titanate (PZT) with polyvinylidene fluoride (PVDF). In this composite, PZT enhances the piezoelectric coefficient, while PVDF contributes flexibility and processability. These composites are widely used in sensors, actuators, and biomedical applications, such as pacemakers [116,117,118,119,120,121,122,123].

### 6.2. KNN-Based Composites

Potassium sodium niobate (KNN) is often combined with polymers like PVDF to create eco-friendly piezoelectric composites. Unlike PZT, KNN does not contain lead, making these composites safer for the environment and suitable for biomedical applications [124,125,126,127,128,129].

### 6.3. BaTiO_3_-Based Composites

Ceramic–polymer composites based on barium titanate (BaTiO_3_) are particularly significant due to their high dielectric constants and effective piezoelectric properties [130,131,132,133]. M. Acosta and collaborators provide a critical review of piezoelectric materials based on BaTiO_3_, discussing the importance of crystallography, piezoelectricity, and strategies to optimize piezoelectric properties through microstructure control and chemical modification. They also examine solid solution systems included in these materials, such as (Ba,Ca)(Zr,Ti)O_3_ and (Ba,Ca)(Sn,Ti)O_3_ [28].

BaTiO_3_, when combined with polymers like PVDF or polyamide family polymers, results in flexible and durable composites used in environmentally sensitive applications like microsensors and wearable devices [33,40,134,135,136,137,138,139]. Barium titanate is also attractive for research as a lead-free piezoelectric material suitable for eco-friendly and biomedical applications [140,141,142,143].

### 6.4. Characterization of Composites

Characterizing ceramic–polymer composites involves measuring piezoelectric coefficients, mechanical stability, temperature response, and analyzing morphology through techniques such as scanning electron microscopy (SEM) and X-ray diffraction (XRD). For instance, in PZT-PVDF composites, uniform dispersion of ceramic nanoparticles in the polymer matrix directly influences the piezoelectric properties of the material [144].

The characterization techniques for ceramic powders involve various methods for analyzing their physical, chemical, and structural properties. These techniques are crucial for understanding the composition, morphology, and behavior of ceramic powders. Some common characterization methods include the following:

Particle size analysis (PS): It is used to evaluate particle size and particle size distribution (PSD), along with other parameters such as zeta potential in a specimen. This technique is applicable for aerosols, emulsions, suspensions, and solid materials [145].

X-ray Diffraction (XRD): This is used to identify the crystalline structure and for qualitative phase analysis of ceramic powders, providing information about crystallinity and phase transformations [146,147].

Scanning Electron Microscopy (SEM): This allows high-resolution images of ceramic powders, providing details about the morphology, size, and distribution of particles [148].

For examples, some of the studies on the microscopic structure of the BaTiO_3_/PMMA composite are presented next.

Studies on composites made by Subhrangsu Dey et al. [149] from barium titanate (BaTiO_3_) particles dispersed in a polymethyl methacrylate (PMMA) matrix highlight the interest in their remarkable optical and dielectric properties. According to several sources [149,150,151,152,153], uniform dispersion of particles in the matrix is essential for maximizing the material’s performance. At a microscopic scale, a homogeneous distribution improves optoelectronic and mechanical properties, avoiding the formation of agglomerates that can negatively affect transparency and dielectric stability.

According to the research [149], the BaTiO_3_/PMMA interface plays a crucial role in transferring physical properties between the ceramic phase and the polymer matrix. Methods such as surface treatments or the use of coupling agents contribute to a stronger bond at the interface, increasing adhesion and reducing the risk of agglomeration. Studies emphasize that the size and shape of the particles influence how they interact with the matrix, with nanoparticles being associated with more efficient dispersion and superior optical properties.

A.M. Ismail and collaborators [154] analyzed the structure of the composites using scanning electron microscopy (SEM) to identify the particles dispersed in the PMMA matrix and evaluate the quality of the interface. Optimal control over the microscopic structure of the composite is necessary to achieve improved material performance in optoelectronic and dielectric applications.

Energy Dispersive X-ray Spectroscopy (EDS or EDX): Elemental chemical analysis of powders, sintered ceramic materials, and composite materials. EDS analysis can perform localized analyses, overlapping spectra, and maps of the elemental chemical composition [155,156].

Transmission Electron Microscopy (TEM): Provides images at a much higher resolution of ceramic powders, allowing detailed analysis of individual particles, crystal defects, and interfaces [157].

Raman Spectroscopy: It is an analytical technique in which scattered light is used to measure the vibrational energy modes of a sample. It can provide both chemical and structural information, as well as the identification of substances through the characteristic “fingerprint” of Raman. This technique extracts information by detecting the Raman scattering from the sample [158].

Fourier Transform Infrared Spectroscopy (FTIR): It is used for analyzing the chemical composition and bonding characteristics of ceramic powders by measuring their infrared absorption spectra [159].

Differential Scanning Calorimetry (DSC) and Thermogravimetric Analysis (TGA): They are used for studying the thermal properties of ceramic powders, including melting behavior, phase transitions, and thermal stability [160].

Brunauer–Emmett–Teller (BET) surface analysis: It is used to determine the specific surface area of ceramic powders, which is important for understanding reactivity and adsorption properties. It is based on measuring the amount of gas (usually N2) adsorbed onto the surface of solids, whether porous or non-porous [161].

Porosity analysis: Various techniques such as mercury porosimetry or gas adsorption methods can be used to quantify the porosity and pore size distribution of ceramic powders [162].

These characterization techniques provide valuable information for optimizing the synthesis processes of ceramic powders, controlling the quality of products, and predicting the performance of materials in various applications.

The characterization techniques for piezoelectric materials involve methods for evaluating their electrical, mechanical, and structural properties. These techniques are essential for understanding the behavior and performance of piezoelectric materials in various applications. These methods may include the following:

Measuring the piezoelectric coefficient: This involves techniques such as measuring the d33 coefficient or the Berlincourt method to measure the piezoelectric coefficients of the material, which quantifies its ability to convert mechanical energy into electrical energy and vice versa [163,164].

Analysis of dielectric properties: Dielectric spectroscopy or impedance analysis is used to study the dielectric properties of piezoelectric materials, including permittivity, loss tangent, and response frequency [165].

Measurement of ferroelectric hysteresis: This method measures the ferroelectric hysteresis loop using techniques such as the Sawyer–Tower circuit or the PUND method to analyze the polarization–electric field behavior of the material [166,167].

Testing of mechanical properties: Techniques such as nanoindentation or dynamic mechanical analysis (DMA) are used to evaluate the mechanical properties of piezoelectric materials, including stiffness, hardness, and elastic modulus [168].

Electrical characterization: This involves techniques such as impedance spectroscopy (EIS), capacitance–voltage (C-V) measurements, and current–voltage (I-V) measurements to study the electrical behavior and performance of piezoelectric materials under different conditions [169].

## 7. Applications of Polymer–Ceramic Piezoelectric Composites

### 7.1. Flexible Sensors and Wearable Electronics

Polymer–ceramic piezoelectric composites are increasingly used in wearable electronics due to their flexibility and ability to generate electrical energy from the mechanical movements of the user. These devices are integrated into smart textiles, fitness bracelets, and other wearable equipment to monitor physiological parameters such as blood pressure, heart rate, and muscle movements [109,170,171].

Flexible sensors are made from polymer composites that include dispersed piezoelectric ceramic phases, providing an adaptable mechanical response and high sensitivity to external stimuli [7,172,173,174]. An example is the use of PVDF in flexible sensors for monitoring blood pressure and detecting mechanical vibrations [175]. Additionally, these composites are used to monitor environmental parameters and detect defects in infrastructure structures, such as bridges and buildings [176,177].

### 7.2. Energy Harvesting

Another important application area for polymer–ceramic piezoelectric composites is energy harvesting from mechanical movements or vibrations [178]. These composites are used in smart infrastructures, such as roads or bridges, where the energy produced by vehicles can be captured and converted into electrical energy [179]. Additionally, wearable devices that utilize piezoelectric composites can transform the energy from body movement into electricity, thus powering sensors or other small devices [143,180,181,182].

Recently, energy harvesting from the vibrations produced by human movements has been a topic of interest, and polymer–ceramic composites have demonstrated high potential in these applications. For example, research has shown that devices based on BaTiO_3_/PVDF can generate enough energy to power small electronic devices, such as LEDs and wireless sensors [183].

## 8. Charge Transfer Models

### 8.1. Theoretical Models

Charge transfer at the interface between the polymer and the ceramic phase is one of the most critical factors influencing the piezoelectric performance of hybrid composites. Theoretical models, such as the percolation model, explain that ceramic particles must form a continuous network within the polymer matrix to allow for efficient charge transfer [184].

In the work [185], Qian Zhang and collaborators present an extension of the hybrid particle–field method to study the self-assembly and percolation behavior of nanocomposites containing block copolymers and nanoparticles. The results indicate that block copolymers can either promote or hinder the formation of the percolation network, depending on their composition. There is an optimal composition that minimizes the percolation threshold, facilitating the formation of a continuous nanoparticle structure. Additionally, modifying the shape of the nanoparticles can further reduce this threshold, thereby enhancing the material’s properties. Other research has demonstrated that the uniform distribution of ceramic nanoparticles can significantly improve the piezoelectric properties of 3-0 and 1-3 composites [186,187,188].

### 8.2. Improving Charge Transfer Through Functionalization

Functionalization of ceramic particles or polymers can enhance charge transfer efficiency. Chemical treatments applied to the surface of the polymer or ceramic phase allow for improved adhesion at the interface between the two phases, leading to better integration of the composites [189,190].

In the work [191], Smaranika Dash and collaborators elucidate the mechanism for enhancing the dielectric and ferroelectric behavior of polymer–ceramic composites through the hydroxylation of the surface of filler ceramic particles (BZT-BCT) incorporated into a PVDF-HFP copolymer matrix. They demonstrate that the use of these hydroxylated fillers in the composites results in a significant improvement in dielectric properties, resulting in a material with superior performance.

Yang Tong and his collaborators studied the influence of a commercial coupling agent on the dielectric properties and microstructure of ceramic–polymer nanocomposites. They found that coating the surface of BaTiO_3_ fillers with a small amount of coupling agent (aminopropyltriethoxysilane) leads to an improvement in dielectric constant, greater resistance to electrical discharge, and increased energy storage efficiency. The results show that the thin layer of coupling agent optimizes the interaction between the ceramic and polymer phases, thus contributing to the enhanced performance of the composite material [67].

Another example is the addition of coupling agents, such as silanes, at the ceramic–polymer interface, which can prevent the formation of voids and improve charge transfer [192,193].

## 9. The Influence of Nuclear Radiation on Hybrid Polymer–Ceramic Piezoelectric Composites

Piezoelectric composites are frequently used in radioactive environments, such as nuclear power plants. Understanding how nuclear radiation affects these materials is essential for developing hybrid piezoelectric materials with high durability. Nuclear radiation can cause degradation and accelerated aging of piezoelectric composites with a polymer matrix, thereby reducing their ability to generate electric charge when subjected to mechanical stresses. The negative effects are felt on both the polymeric and piezoelectric phases, leading to a decrease in the mechanical and piezoelectric performance of the material.

The types of nuclear radiation that act on materials are as follows:

- Alpha Radiation (α): Particles composed of two protons and two neutrons, which have a low penetration capacity.
- Beta Radiation (β): High-energy electrons or positrons, with a greater penetration capacity than alpha radiation, but still limited.
- Gamma Radiation (γ): High-energy electromagnetic radiation, characterized by a high potential for penetration.
- Neutrons: Very penetrating neutral particles capable of producing significant changes in the structure of materials.

Hybrid polymer–ceramic piezoelectric composites are increasingly investigated for applications in harsh environments, including those involving nuclear radiation. The effects of radiation exposure can be divided into two main categories: (1) changes in electrical properties due to ionization and (2) degradation of mechanical or structural integrity caused by radiation-induced defects [194].

Studies have shown that gamma radiation exposure up to certain doses (e.g., 10–100 kGy) can lead to a slight decrease in the piezoelectric coefficient (d_33_) of composites. This reduction is attributed to radiation-induced chain scission in the polymer matrix, leading to reduced stress transfer between the matrix and ceramic filler. For instance, PVDF-based composites exhibited a 10–15% reduction in d_33_ after exposure to 50 kGy of gamma radiation, while ceramic fillers like BaTiO_3_ retained their intrinsic piezoelectric properties due to their higher radiation resistance [195,196,197,198,199,200].

Neutron radiation primarily affects the ceramic phase due to its interaction with crystal lattice atoms, leading to displacement damage. For example, in BaTiO_3_ or PZT-based composites, neutron fluences of 1012–1014 n/cm^2^ have been reported to induce minor shifts in dielectric constants due to defect accumulation in the ceramic lattice. However, the polymer matrix may partially shield the ceramic phase, mitigating these effects [201,202].

The practical performance of hybrid composites under nuclear radiation exposure suggests that these materials are suitable for moderate radiation environments, such as space exploration and nuclear power plants. However, applications in high-radiation areas require additional measures, such as radiation-hardening treatments or protective coatings.

In the paper [203], Traian Zăhărescu studied the effects of radiation on various polymer composites with the aim of expanding the applications in which they can be used. The examples presented in this paper provide a starting point for future studies.

The effects on ceramic–polymer composites manifest both on the polymer matrix and on the piezoelectric phase [197,204]:

Degradation of Polymer Chains: Radiation, particularly gamma rays and neutrons, can break the chemical bonds in the polymer structure, leading to a decrease in mechanical and physical properties (e.g., elasticity and tensile strength) [205].

Changes in Polymer Crystallinity: In the case of piezoelectric polymers like PVDF, exposure to radiation can alter the degree of crystallinity, affecting its ability to generate electric charge [206,207].

Ionization of the Polymer: Gamma radiation can induce ionization in the matrix, affecting the electrical conductivity of the material and causing a loss of piezoelectric performance [208].

In the paper [209], Jassim M. Yaseen studied the behavior of BaTiO_3_ following irradiation and the effects of fast neutrons and gamma radiation on the microstructure and dielectric properties.

In the study [210] conducted by M.M. Atta and his collaborators, the fabrication of polyvinylidene fluoride–barium titanate (PVDF-BT) films with varying amounts of polyaniline (PANI) was analyzed using the solution casting method. The PANI content varied from 0% to 2% by weight. The structural and dielectric properties of the films were examined, including those of a sample exposed to electron beam (EB) irradiation at different doses (0, 10, 20, and 30 kGy).

After the irradiation, the following observations were made.

Structural Modifications: The addition of PANI in PVDF-BT increased the amount of the β-phase, confirmed by X-ray diffraction (XRD) analysis and Fourier-transform infrared (FTIR) spectroscopy. EB irradiation further improved the β-phase by inducing the transition from the α-phase, while also causing the amorphization of barium titanate (BT) particles.

Dielectric Properties: The addition of PANI led to an increase in the dielectric constant (ε’) and the loss tangent (tanδ), indicating improved dielectric performance. However, EB irradiation caused a decrease in these values, most likely due to the reduced level of polarization in the irradiated samples.

Electrical Conductivity: The alternating current (σac) conductivity increased with the rising PANI content in the composites, suggesting better electrical conductivity. In contrast, increasing the EB dose led to a decrease in σac, likely due to the reduction in mobile ions and lattice tension.

Additional Structural Modifications: Both PANI doping and EB irradiation produced significant structural changes, including a reduction in lattice tension and the number of mobile ions in the material.

The combined effects of PANI doping and EB irradiation on the PVDF-BT composites demonstrated significant changes in phase composition, dielectric behavior, and electrical properties, which are essential for optimizing the material’s performance in applications such as sensors and energy harvesting devices.

R. A. Zaghlool et al. [211] utilized polyacrylamide (PAM) as a matrix material to prepare samples of nanocomposites loaded with different mass fractions (1, 3, 5, 7, and 15%) of barium titanate (BaTiO_3_) using the casting method. The PAM sample with 7% BaTiO_3_ was irradiated with gamma radiation at a dose of 200 Gy to investigate the impact on the structure and optical and dielectric properties.

The results showed that the BaTiO_3_ nanoparticles were well incorporated into the PAM matrix, and the crystal size decreased due to dispersion in the matrix, as confirmed by Fourier-transform infrared (FTIR) spectroscopy and X-ray diffraction (XRD) analyses.

Additionally, the incorporation of 7% BaTiO_3_ in PAM increased the dielectric permittivity from 1.07 to 2.44 (at 100 kHz), with a slight increase in the loss factor from 0.015 to 0.020. On the other hand, gamma irradiation reduced the crystal size compared to the unirradiated sample, increasing the dielectric permittivity to 2.89 and causing a slight decrease in the loss factor (0.019) at 100 kHz.

These modifications indicate that both the filling with BaTiO_3_ nanoparticles and exposure to gamma radiation significantly influence the structure and electrical properties of composite materials, offering potential advantages in optoelectronic and dielectric applications.

To enhance the radiation resistance of piezoelectric composites based on barium titanate (BaTiO_3_) and polymers, various strategies for the chemical modification of the components can be applied to reduce radiation-induced degradation and improve material stability. Various studies have been conducted in this regard; for example, in the paper [212], Udhay Sundar et al. discuss surface treatments of BaTiO_3_ nanoparticles with coupling agents to enhance compatibility with the polymer matrix and reduce defects that may lead to degradation under radiation exposure. In the paper [213], Liming Liu et al. discuss the use of doping agents (DET) to increase durability and piezoelectric performance, with BaTiO_3_ nanoparticles modified with DET showing improved dispersion in the PVDF matrix, enhancing both mechanical stability and radiation resistance of the composite. This modification alleviates aggregation and improves the interfacial bonding, leading to better durability under cyclic stress and radiation.

Modification of BaTiO_3_ Particle Surfaces: Treating the surface with coupling agents, such as silanes or organic phosphates, can improve the interfacial compatibility between ceramic particles and the polymer matrix. These surface treatments help achieve uniform dispersion of the particles in the matrix and can reduce the formation of defects that could act as initiation points for radiation-induced degradation. Thus, the composite’s radiation resistance can be improved by reducing susceptibility to cracking and fracturing under radiation exposure [214].

Incorporation of Radiation Stabilizers: Adding chemical stabilizers to the polymer matrix can increase the radiation resistance of the composites. These substances, such as free radical stabilizers, can prevent or slow down the degradation process of the polymer under the action of ionizing radiation. Stabilizers work by capturing free radicals formed during radiation exposure, thereby reducing the negative effects on the mechanical and piezoelectric properties of the material [215,216].

Controlled Crosslinking of the Polymer Matrix: The crosslinking process can enhance the durability of the composites against radiation by forming additional chemical bonds within the polymer structure. Crosslinking makes the matrix more resistant to degradation and reduces susceptibility to decomposition under radiation exposure, thereby improving the mechanical and functional properties of the composite [217].

These techniques help in developing radiation-resistant ceramic–polymer composites, which are useful in applications in high-radiation environments, such as in space equipment or the nuclear industry.

Molecular Grafting: This involves attaching side chains of polymers or chemical compounds directly onto the main polymer chain to enhance chemical stability under radiation conditions. Aromatic compounds, siloxanes, or polydimethylsiloxanes can be used for grafting [218]. Aromatic compounds are polymers containing aromatic structures (e.g., benzene) that can uniformly distribute absorbed energy, potentially preventing or reducing the cleavage of polymer chains. Siloxanes and polydimethylsiloxanes (PDMSs) [219] are compounds with high radiation resistance, especially used in composites working in extreme environments (including space). Grafting these onto polymer chains can add durability and stability.

Polymers with intrinsic chemical stability, which have a natural higher resistance to radiation due to their chemical structure, are polymers containing fluorine atoms and are less susceptible to cleavage and crosslinking. They are used in applications in nuclear radiation environments. Examples in this category include polytetrafluoroethylene (PTFE), which exhibits exceptional stability in harsh environments and radiation, and fluorosilicone elastomers with resistance to radiation and aggressive chemical agents.

## 10. Perspectives

The challenges in developing piezoelectric composites include the following:

- Achieving materials with good compactness, overcoming the difficulties of proper integration between piezoelectric ceramic phases and the polymer matrix, primarily due to differences in the mechanical and thermal properties of the two materials.
- Optimizing the composite structure to achieve suitable values for piezoelectric coefficients and electromechanical coupling, enhancing energy transfer efficiency.
- Manufacturing structures with precise dimensions, controlled microstructures, and high uniformity using advanced technologies at acceptable costs.
- Ensuring product reliability, as resistance to mechanical stress and repeated usage cycles can be limited, affecting long-term reliability.

Potential solutions could include the following:

- Developing innovative hybrid materials with improved compatibility between the composite phases.
- Utilizing advanced manufacturing technologies to ensure uniform structures.
- Optimizing the composite’s geometric design, such as 1-3 or 2-2 dimensional structures, for better piezoelectric performance.
- Applying protective layers and surface treatments to enhance durability and resistance to harsh environments.

## 11. Conclusions

The study of piezoelectric ceramic–polymer composite materials emphasizes their importance in various modern applications due to their unique properties, which combine the high piezoelectric sensitivity of ceramics with the flexibility and processability of polymers. This synergy between the ceramic and polymer phases has enabled the development of advanced materials suitable for use in fields such as energy harvesting, biomedical devices, and flexible sensors.

Current research focuses on optimizing these composites by using piezoelectric polymers like PVDF and combining them with lead-free ceramics (BaTiO_3_, KNN), thus providing eco-friendly and efficient solutions. Additionally, recent advances in processing technologies, such as 3D printing and the functionalization of ceramic–polymer interfaces, have contributed to improving the electrical and mechanical performance of the materials. The addition of polymers to piezoelectric composites serves to impart flexibility and impact resistance to the materials. Polymers such as PVDF (polyvinylidene fluoride) and PTFE (polytetrafluoroethylene) are favored for their ability to enhance the processability of materials, especially in wearable devices and sensors. Polymers allow deformation under mechanical stress, and the molecular alignment of their chains can enhance the piezoelectric properties.

Piezoelectric composites of types 3-0, 1-3, and 2-2 offer a wide range of structures tailored to the specific needs of applications, from vibration sensors to energy conversion devices. In particular, the use of coupling agents and nanomaterials has led to significant improvements in the overall performance of composites, especially regarding charge transfer and mechanical stability.

Ceramic piezoelectric materials, such as PZT (lead zirconate titanate) and BaTiO_3_ (barium titanate), are known for their high piezoelectric coefficients, making them very effective in generating electric charge under the influence of mechanical force. However, these materials are fragile and have limitations in applications requiring flexibility. For example, the piezoelectric coefficient of PZT can vary between 300 and 700 pC/N, depending on the exact composition and processing conditions.

Nuclear radiation, especially gamma rays and neutrons, can cause breaks in polymer chains and defects in the crystalline structure of piezoelectric materials. These defects reduce the material’s ability to generate electric charge, affecting its piezoelectric and mechanical performance. In piezoelectric materials, depolarization can occur as a result of gamma radiation or neutrons, leading to the loss of piezoelectric properties. Exposure to radiation can accelerate the aging of piezoelectric materials, resulting in a rapid decrease in piezoelectric sensitivity and flexibility. Microcracks that appear in the structure of materials also contribute to premature degradation and failure of functionality.

Polymers can be enhanced by doping with stabilizers that prevent radiation-induced degradation. For example, the use of antioxidants can prevent the oxidation of polymers, while UV or gamma stabilizers absorb radiation energy and dissipate it as heat, preventing the breaking of polymer chains. Attaching side chains or creating chemical bonds between polymer chains can increase resistance to radiation. The use of compounds such as siloxanes and polydimethylsiloxanes (PDMS) adds durability to materials, being particularly useful in extreme applications, such as space. Fluorinated polymers, such as PTFE, are recognized for their exceptional resistance to radiation and harsh conditions. They are preferred in applications involving prolonged exposure to nuclear radiation due to their natural resistance to chemical and thermal degradation.

Modern manufacturing techniques, such as 3D printing, allow for the creation of complex structures and precise integration of ceramic and polymer phases. Methods such as stereolithography or Direct Ink Writing provide the opportunity to fabricate customized piezoelectric devices, optimizing both internal structure and mechanical and electrical properties.

Chemical treatments at the polymer–ceramic interface, such as using coupling agents, improve adhesion and charge transfer between the two phases. This reduces the risk of delamination and increases the efficiency of the composites.

As modern technologies continue to evolve, ceramic–polymer piezoelectric composites have the potential to revolutionize fields such as wearable devices and smart infrastructures. Thus, the participation of polymer materials in the structure of composites remains essential for the development of new sustainable and efficient technologies capable of meeting the growing demands of modern society. Piezoelectric composites are increasingly used in devices that capture energy from mechanical vibrations or human movements, converting it into electricity to power sensors or small electronic devices.

These applications are particularly relevant for smart infrastructures and portable devices. The flexibility and durability of piezoelectric composites make them ideal for use in medical sensors, implants, or portable health monitoring devices. Sensors based on piezoelectric composites can monitor physiological parameters and provide real-time feedback.

The use of radiation-resistant and harsh condition-resistant materials opens new opportunities in nuclear and space applications, where piezoelectric composites can be integrated into critical equipment that must operate in hostile environments without performance loss.

## Figures and Tables

**Table 1:** Comparative properties of composites with piezoelectric materials [86,87,88,89,90,91].

Property	Polymer Matrix Only	Ceramic Filler Only	Polymer/Ceramic Composite	Remarks
**Piezoelectric Coefficient (** ***d*_33_, pC/N)**	Negligible (<1)	High (100–500)	Intermediate (10–100)	Composite performance depends on filler dispersion and interfacial bonding.
**Flexibility**	High	Low	Moderate	Composites retain some flexibility, suitable for wearable applications.
**Dielectric Constant**	Low (2–4)	High (100–1000)	Intermediate (10–100)	Enhanced dielectric constant improves energy storage but introduces challenges in uniformity.
**Density (g/cm^3^)**	Low (0.9–1.2)	High (4–6)	Moderate (1.5–3)	Composites strike a balance between weight and performance.
**Processability**	Excellent	Challenging	Good	Composites are easier to process compared to pure ceramics while retaining useful properties.
**Thermal Stability**	Moderate (150–200 °C)	High (>1000 °C)	Moderate (200–300 °C)	Limited by the polymer matrix but sufficient for most applications.
